# Insights From the Nihon Housou Kyoukai’s Virtual Reality–Based Social Interaction Television Program “Project Aliens” for Adolescents With Psychiatric Disorders: Single-Center Case Series Study

**DOI:** 10.2196/74401

**Published:** 2025-05-30

**Authors:** Junichi Fujita, Mizuho Takayama, Emi Kamono, Satoru Shinoda, Hiroyuki Yamaguchi, Tomoko Moroga, Mio Ishii, Tomoyuki Miyazaki

**Affiliations:** 1 Department of Child Psychiatry Yokohama City University Hospital Yokohama Japan; 2 Department of Biostatistics Graduate School of Medicine Yokohama City University Yokohama Japan; 3 Department of Psychiatry Yokohama City University School of Medicine Yokohama Japan; 4 Center for Research and Industry-Academia-Government Collaboration Yokohama City University Yokohama Japan

**Keywords:** virtual reality, social interaction, adolescents, mental health, case series, peer support, therapeutic facilitation

## Abstract

**Background:**

Virtual reality (VR) technology is emerging as a tool in mental health care, providing a safe space for social interaction and therapeutic engagement. A social VR-based television program broadcast on Japanese public television offers a virtual environment where adolescents with mental health challenges can engage in peer support using alien avatars, reducing barriers to communication and encouraging emotional expression.

**Objective:**

This case series aimed to document the psychological trajectories of adolescents with psychiatric disorders participating in a social VR-based television program.

**Methods:**

A single-center case series was conducted with 3 adolescents with psychiatric disorders (aged 15, 18, and 19 years) who participated in the social VR-based television program. The study focused on examining patient-reported outcomes (PROs), including psychological measures and qualitative experiences, and clinical observations across program participation and broadcast viewing. Psychological measures, including the Japanese versions of the 3-item Short-Form University of California, Los Angeles Loneliness Scale (UCLA-LS3-J SF-3), the 14-item Resilience Scale, short form (RS-14), and the 9-item Patient Health Questionnaire (PHQ-9), were assessed at 3 time points: baseline, prebroadcast, and postbroadcast. Qualitative analysis of participant dialogue explored themes of self-disclosure, emotional expression, and social dynamics.

**Results:**

Participants showed improvements in loneliness, resilience, and depressive symptoms after participating in the social VR-based program, as indicated by psychological measures and PROs. Qualitative analysis suggested that the structured facilitation embedded in the program enabled participants to express positive and negative emotions, promoting self-reflection and mutual support.

**Conclusions:**

This case series suggests that structured social VR programs can provide a supportive platform for emotional exploration and psychological growth among adolescents with psychiatric disorders. The combination of avatar-based interaThis case series suggests that structured social VR-based programs can provide a supportive platform for emotional exploration and psychological growth among adolescents with psychiatric disorders. The combination of avatar-based interaction and therapeutic facilitation may offer a novel approach to engaging young people in mental health care, particularly during waiting periods for traditional psychiatric services.ction and therapeutic facilitation may offer a novel approach to engaging young people in mental health care, particularly during waiting periods for traditional psychiatric services.

## Introduction

Virtual reality (VR) technology has emerged as an innovative therapeutic tool in mental health care, particularly for individuals with social anxiety, depression, and related conditions [1]. Although VR-based exposure therapy and cognitive behavioral interventions have been well documented [2], VR environments are increasingly being used as therapeutic spaces providing safe gathering places for group therapy settings or social skill training for individuals with mental health challenges. However, the therapeutic potential of social VR environments—spaces where users can interact through avatars—remains largely unexplored [3,4]. In Japan, various VR-based social participation programs have been implemented to address school refusal—a significant educational challenge in which students do not or cannot attend school due to psychological, emotional, physical, or social reasons, excluding illness, financial difficulties, or COVID-19–related concerns—affecting over 240,000 students in 2021 [5].

Building on these developments, Japan’s public broadcaster (Nihon Housou Kyoukai [NHK]) developed *Project Aliens*, an innovative television program that provides a VR environment where adolescents interact through alien avatars [6]. Participants communicate in a virtual space using alien avatars, which provides a psychologically safe medium for self-disclosure and fostering connections based on shared experience. Therefore, the *Project Aliens* series offers a compelling model for VR-based social experiments with clinical relevance. By lowering barriers to self-expression and creating a supportive VR-based environment, the series aims to help participants confront personal and interpersonal challenges in ways that may be less achievable in conventional therapeutic settings. This social VR-based approach aligns with broader explorations into the metaverse’s potential for mental health care, where users can interact within a customizable, immersive environment that offers emotional safety and flexibility, critical for therapeutic engagement among young people. Each episode of *Project Aliens* explores a unique theme, including social stigma, family dynamics, and self-identity. Thus, it can be recommended as a tool for fostering emotional growth and social connection.

Among children and adolescents, barriers to seeking and accessing mental health services remain significant challenges, with the most prominent barriers being social stigma and individual factors, such as limited mental health knowledge [7]. Although peer support has shown promise in promoting recovery among young people with mental health conditions [8-10], traditional face-to-face interventions often face barriers related to social anxiety and stigma [11]. Social VR-based platforms may address these challenges by enabling anonymous, avatar-based interactions [12,13]. However, the psychological impact of structured, VR-based peer support programs for adolescent psychiatric patients remains poorly understood. To address this gap, this case series investigated the impact of participating in *Project Aliens* on adolescents’ psychological well-being, specifically, by examining changes in loneliness, depression, resilience, and patterns of emotional expression during VR-based interactions.

## Methods

### Study Design

This single-center, observational case series evaluated the psychological effects of participation in *Project Aliens*, a Japanese public television program using social VR-based peer dialogue among adolescents with psychiatric disorders.

Participants meeting the inclusion and exclusion criteria were enrolled after providing consent for both program participation and study involvement. Changes in psychological indicators were assessed through data collection before recording and after broadcast.

### Participants

Eligible participants were child and adolescent patients with psychiatric disorders receiving care at Yokohama City University Hospital who met the following criteria inclusion criteria:

- Participants must be aged 11-19 years at the time of consent.
- Participants must be in a stable mental health condition, as assessed by their primary care physician.
- Both the participant and their family must fully understand the purpose of the NHK program
  Project Aliens
  and agree to appear on the show.
- Written consent for participation in the study must be obtained.

The exclusion criteria were as follows:

- The presence of intellectual disabilities that may interfere with understanding the program or study
- Imminent risk of self-harm or suicide
- Suspected cases of abuse by family members
- Severe depression, as indicated by a score of ≥21 in the 9-item Patient Health Questionnaire (PHQ-9)
- Deemed unsuitable for participation based on clinical judgment by the primary care physician

### Recruitment

Participants were recruited from the Department of Child Psychiatry at Yokohama City University Hospital. Between March 14 and April 12, 2024, a total of 15 adolescent patients receiving outpatient care were invited to participate. After follow-up reminders, 10 (67%) patients expressed interest and completed a program-specific questionnaire designed to assess their motivation for participation and potential for engagement in the virtual environment.

A sample size of 3 participants was prespecified based on both production requirements and methodological considerations. Although some previous episodes of *Project Aliens* had featured 4 participants, for this specific episode, the production team determined that 3 participants would be optimal to balance participant interaction with the facilitation role of the Moon Rabbit character. This decision ensured sufficient dialogue depth and narrative clarity for viewers, while maintaining appropriate pacing for the therapeutic journey.

The final selection of participants was conducted collaboratively between clinical staff and NHK producers, with priority given to 3 primary criteria, as documented in preproduction meetings: (1) participant motivation for virtual interaction rather than specific demographic attributes, (2) communication capabilities that would enable meaningful dialogue within the virtual environment, and (3) complementary personality characteristics or “interpersonal variability” that would facilitate dynamic group interaction. Although clinical considerations ensured participant safety and appropriateness for the program, the production team prioritized selecting participants who demonstrated genuine interest in connecting with others in the virtual environment.

From a methodological standpoint, this case series was conceptualized as an in-depth exploration of individual psychological trajectories rather than a study aimed at statistical generalization, making the small sample size appropriate for the detailed qualitative analysis planned. The selected participants included 1 (33%) male and 2 (67%) females, aged 15, 18, and 19 years, respectively, at the time of filming.

### Program Description

This study documented the participants’ experiences during their involvement in *Project Aliens*, a 45-minute late-night special program on Japanese public television. Episode 8 of the series, titled “A Healing Field Trip,” was recorded on July 13-14, 2024, and broadcast on October 27, 2024. Participants joined the *Project Aliens* virtual world remotely from their homes, connecting through personal computers rather than VR headsets.

**Figure 1:**
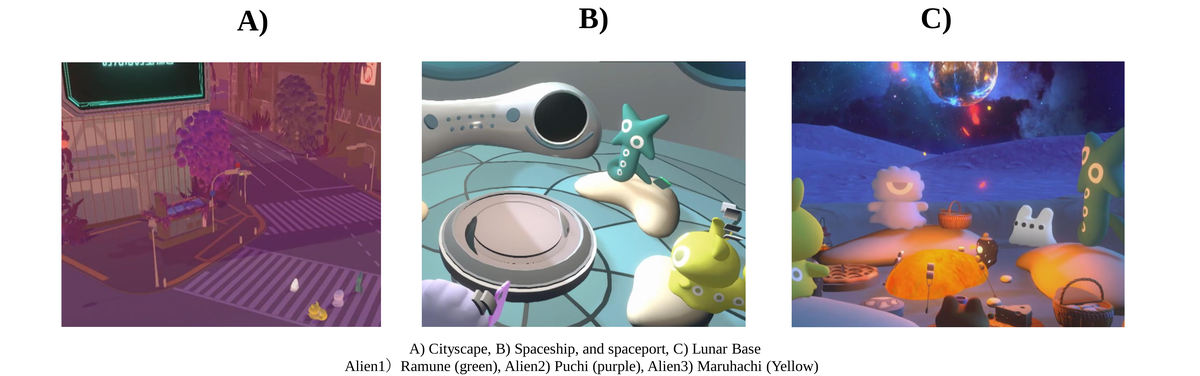
Sequential stages of the VR-based therapeutic journey. The figure depicts 3 sequential therapeutic environments: (A) Cityscape: designed to facilitate initial collaboration and trust formation; (B) Spaceship and Spaceport: structured for deep reflection through narrative sharing; and (C) Lunar Base: focused on personal growth and future orientation. Moon Rabbit (white figure) functions as the therapeutic facilitator, guiding structured activities and maintaining psychological safety throughout the program. This facilitation is crucial in creating a supportive environment that enables meaningful participant interaction and emotional expression. Alien 1 is named Ramune, colored green; alien 2 is named Puchi, colored purple; and alien 3 is named Maruhachi, colored yellow. VR: virtual reality.

The program offered a VR-based journey through 3 distinct stages: (1) Cityscape, (2) Spaceship and Spaceport, and (3) Lunar Base, as shown in Figure 1A-C. This staged progression—from an open urban setting to an enclosed spaceship and, finally, to an expansive lunar landscape—was designed to gradually foster trust, emotional connection, and self-disclosure. In particular, the enclosed spaceship setting encouraged focused conversations and deeper engagement, whereas the openness of the moon symbolized emotional release and perspective expansion, reinforcing participants’ psychological progression.

This approach was rooted in the belief that meaningful dialogue and the breakdown of preconceptions require a carefully structured process of interaction. Each stage was designed to not only support psychological transition but also create an environment where participants could engage in progressively deeper conversations, while feeling psychologically safe.

The virtual environment was created by an artist renowned for distinctive illustrations, 3D artwork, and youth-oriented video projects that have received national and international recognition [14]. To further support participant comfort and engagement, the program incorporated environmental audio elements, such as ambient nature sounds and underwater acoustics, aimed at fostering an immersive, calming atmosphere. These auditory cues were included to reinforce a shared sense of presence among participants and elevate their perception of a unique, transformative experience.

Throughout the program, the participants’ journey was facilitated by a virtual navigator named Moon Rabbit, portrayed in real time by a professional actress. Moon Rabbit guided the 3 participants on their virtual trip to the moon. Unlike a prescripted artificial intelligence (AI) system, Moon Rabbit engaged dynamically with the participants, responding to their interactions and guiding them through each stage of the journey. Its role was carefully designed to foster psychological safety, manage psychological transitions, and encourage peer support and self-reflection. This approach was developed in collaboration with mental health professionals to ensure that it aligned with therapeutic principles. Moon Rabbit is the white character shown in Figure 1.

#### Cityscape: Discovering Life Through Each Other’s Treasures

The journey began in a virtual recreation of Shibuya City in Tokyo, a lively urban environment popular among young people. The participants collaborated in a treasure hunt to locate hidden “treasures” within the city. This activity encouraged teamwork and provided an initial opportunity for participants to share personal insights, building trust and familiarity within the group.

The urban setting was intentionally chosen as a familiar yet dynamic social space where participants could ease into interactions. For those from rural areas, it introduced them to a different social environment. The open nature of the cityscape provided a sense of exploration and informal engagement, helping participants gradually become comfortable in the setting before transitioning to deeper discussions.

#### Spaceship and Spaceport: Discovering We Are Not Alone

In this stage, the participants boarded a spaceship heading to the moon. Here, they encountered narratives from former participants, or “senior aliens,” who shared their transformative journeys. The participants also shared personal photos and discussed their daily struggles, fostering deeper self-reflection and mutual understanding.

The spaceship, a deliberately enclosed environment, was designed to foster focused, structured conversations. The transition from an open cityscape to a confined space created a sense of intimacy, encouraging self-disclosure and deeper emotional engagement. At the spaceport, participants undertook the final task of the journey: writing letters to themselves. These self-reflective letters explored past challenges, present struggles, and aspirations for the future. Reading these letters aloud allowed the participants to express self-compassion and find closure. This transitional stage, just before reaching the moon, offered the participants an opportunity to consolidate their thoughts and prepare for the final phase of the journey.

#### Lunar Base: Sharing Painful Pasts, Stepping Toward the Future

The final stage took place on the moon, representing a safe space for introspection and transformation. The participants reflected on their experiences, shifting the focus from their past to envisioning their future. This phase highlighted the progress they had made throughout the journey.

The moon was chosen for its vast, open landscape, symbolizing emotional release and perspective expansion. In stark contrast to the confined spaceship, the moon represented liberation and psychological progression. The openness of the moon encouraged the participants to engage in forward-looking discussions, marking a symbolic transition from structured reflection to envisioning new possibilities in their lives.

### Case Presentations

The following cases describe the 3 participants who engaged in the VR-based journey described before. Each participant’s avatar appearance is indicated in Figure 1.

#### Case 1: Alien 1 (Ramune)

A 19-year-old male (Ramune) grew up in a socioeconomically disadvantaged family with significant mental health comorbidities. His father had bipolar disorder, his mother experienced depression and asthma, and his sister was diagnosed with autism spectrum disorder (ASD) and bipolar disorder. Diagnosed with ASD and a learning disability at age 8 years, the patient demonstrated communication difficulties, experienced persistent bullying, and subsequently developed severe social anxiety disorder, leading to extended periods of social withdrawal. Prolonged periods of housebound behavior further isolated him. Telemedicine during the COVID-19 pandemic enabled him to reconnect with psychiatric care, leading to improvements in his emotional stability. By the time of his participation, Ramune was attending a vocational training facility and a youth support center but remained hesitant about interpersonal interactions. Ramune is the green character in Figure 1.

#### Case 2: Alien 2 (Puchi)

A 15-year-old female (Puchi) presented with a history of exposure to high academic pressure. The patient developed major depressive disorder with psychotic features at age 11 years, coinciding with preparation for private school entrance examinations. Subsequently, she developed body dysmorphic disorder, exacerbated by social media exposure, which led to familial discord and suicidal behavior. Despite progress, Puchi continued to experience fatigue and emotional fragility. Puchi is the purple character in Figure 1.

#### Case 3: Alien 3 (Maruhachi)

An 18-year-old female (Maruhachi) presented with a history of severe academic and familial stressors. By age 15 years, her rigorous schedule led to severe weight loss, panic attacks, and eventually hospitalization for generalized anxiety disorder and an eating disorder. After 1 month of inpatient nutritional rehabilitation and antidepressant treatment, she regained physical health but continued to struggle with social isolation and reintegration into school life. Maruhachi is the yellow character in Figure 1.

### Outcome Measures

#### Primary Outcome Measure

The primary outcome measure in this study was the longitudinal progression of each participant, assessed from baseline (study registration) through prebroadcast and postbroadcast evaluations. This case series aimed to document individual trajectories by examining changes in self-reported experiences (patient-reported outcomes [PROs]) and clinical observations.

To capture subjective experiences, PROs included self-reported loneliness, resilience, and depressive symptoms, as well as open-ended reflections on interpersonal relationships, emotional states, and coping strategies. These qualitative data were supplemented with psychiatrist assessments, based on routine consultations and medical records, to provide a clinical perspective on participants’ psychological status.

#### Secondary Outcome Measures

##### Psychological Measures

The Japanese version of the 3-item Short-Form University of California, Los Angeles Loneliness Scale (UCLA-LS3-J SF-3) was used. The UCLA-LS3-J SF-3 is a concise tool developed for the rapid assessment of loneliness. This scale has been validated in various contexts, including among mothers with infants and toddlers, demonstrating its versatility and robustness in capturing loneliness across different populations. The 3-item version is derived from the 10-item version, with a high correlation to the original UCLA Loneliness Scale version 3. It has shown adequate reliability and validity in Japanese studies, making it particularly suitable for brief interventions [15]. The total score on the UCLA-LS3-J SF-3 ranges from 3 to 9.

The 14-item Resilience Scale, short form (RS-14) is a validated tool for assessing resilience, which is conceptualized as the capacity to recover from adversity. Its reliability and validity have been established in Japanese populations, including its psychometric equivalence to the original RS. The short form reduces respondent burden, while maintaining robust internal consistency and test-retest reliability [16]. The total RS-14 score ranges from 14 to 98.

The PHQ-9 was selected because assessing treatment efficacy through PHQ-9 score changes is commonly recommended and widely accepted in clinical research, including in Japan [17]. The PHQ-9, a self-administered questionnaire comprising 9 items, evaluates the presence and severity of depressive symptoms within the past 2 weeks, based on *Diagnostic and Statistical Manual of Mental Disorders, Fourth Edition* (DSM-IV) criteria for major depressive disorder. The total PHQ-9 score ranges from 0 to 27, with higher scores indicating more severe depressive symptoms.

Assessments were conducted at 3 time points: baseline (study enrollment), interim (4-10 days pre-recording), and final (4-10 days postbroadcast). For all evaluations, participants completed the questionnaires at home on paper. The completed questionnaires were then either returned during their next outpatient visit or sent by mail. This schedule was consistently applied for all 3 measures to ensure data reliability and consistency.

##### Speech Data

Speech data were collected from participants during the program’s metaverse-based interactions.

### Data Analysis

To analyze the primary outcome, we created a structured summary table outlining the longitudinal progression of each participant from baseline (study registration) to postbroadcast. This table summarized participants’ qualitative self-reports at 3 time points (baseline, prebroadcast, and postbroadcast), capturing their experiences of loneliness, emotional responses, and coping strategies. As an ad hoc analysis, a case-by-case qualitative analysis was conducted to further explore these individual trajectories. This analysis focused on participants’ self-reported reflections on loneliness, emotional states, and coping strategies, based on responses to the following open-ended questions:

- During the past 10 days, have you felt lonely in your relationships with friends, family, or society?
- How did you feel at that moment?
- How did you cope with this feeling of loneliness?

Thematic patterns were categorized based on predefined domains aligned with the psychological scales UCLA-LS3-J SF-3, RS-14, and PHQ-9. The identified themes included (1) changes in perceived loneliness (eg, descriptions of social isolation or re-engagement with peers), (2) changes in resilience and shifts in coping strategies (eg, engagement in self-reflection, use of peer support, and (3) emotional responses to program participation (eg, depressive state, expressions of hope, anxiety, or relief). These thematic categories were aligned with predefined psychological constructs measured by the (1) UCLA-LS3-J SF-3 (loneliness), (2) RS-14 (resilience), and (3) PHQ-9 (depressive symptoms). This alignment was achieved by examining the semantic content of participant narratives in relation to established dimensions of the respective psychological scales. Two independent researchers performed coding, and discrepancies were resolved through discussion. In addition, clinical observations from psychiatrists’ records were reviewed to assess changes in participants’ social engagement and emotional regulation. The goal was to identify patterns of psychological adaptation throughout the program.

To analyze secondary outcome measures, changes in pre- and postintervention scores were calculated for the psychological measures, including the UCLA-LS3-J SF-3 (loneliness), the RS-14 (resilience), and the PHQ-9 (depressive symptoms). Descriptive statistics were used to summarize individual trajectories and group-level trends across the 3 assessment points: baseline, prebroadcast, and postbroadcast. Given the small sample size, statistical significance testing was not performed, and results were interpreted based on individual-level changes rather than inferential statistics.

Exploratory sentiment analysis of participants’ utterances was conducted to capture patterns of emotional expression. Words were categorized into positive and negative terms, and their frequencies were counted to assess emotional dynamics across different stages of the program (Cityscape, Spaceship and Spaceport, Lunar Base). Initially, text segmentation and morphological analysis were attempted using MeCab, a widely used Japanese text analysis tool. However, existing sentiment dictionaries did not adequately capture the context-specific emotional expressions in this study. Therefore, a custom lexicon was developed to classify words into sentiment categories. The distribution of emotional expressions was analyzed across different program stages (Cityscape, Spaceship and Spaceport, and Lunar Base) to identify shifts in emotional dynamics. The frequency of positive and negative words was calculated, and sentiment distributions were aggregated for each scene to illustrate emotional transitions across the program’s stages. The analysis was performed using R version 4.4.2 (R Foundation for Statistical Computing), with custom scripts using the *dplyr*, *ggplot2*, and *stringr* packages for text processing and visualization. Sentiment classification was performed using a stepwise approach to systematically categorize words and phrases based on their emotional valence. The process involved 3 steps: removal of neutral elements, sentiment categorization, and validation and refinement.

Sentiment classification was performed using a stepwise approach to systematically categorize words and phrases based on their emotional valence. The process involved 3 steps: removal of neutral elements, sentiment categorization, and validation and refinement.

First, neutral elements, such as proper nouns (eg, place or object names), conjunctions, auxiliary verbs, and particles were identified and excluded from the analysis to focus on emotionally meaningful content. Second, the remaining words and phrases were categorized into 3 sentiment categories: positive, negative, or neutral. Positive expressions included words indicating happiness, encouragement, or social connection (eg, fun, excited, relieved), whereas negative expressions encompassed terms associated with distress, anxiety, or isolation (eg, worried, lonely, frustrated). Ambiguous words (eg, different, interesting) were assessed in context before classification. Additionally, commonly used expressions that do not convey strong emotional valence, such as acknowledgments and greetings, were also categorized as neutral. For instance, frequently used words, such as hello and indeed, appeared frequently in the dataset but lacked a clear positive or negative emotional connotation. Finally, to ensure consistency and accuracy, the primary author and co-author (MT) carefully reviewed the classification together. When ambiguous terms were encountered, they analyzed the recorded broadcast content to determine the appropriate classification. This collaborative analysis considered contextual factors, including (1) the surrounding linguistic context within the utterance; (2) the participant’s tone and delivery, as observed in the broadcast; (3) the narrative arc of the participant’s journey within the program; and (4) the clinical understanding of adolescent emotional expression in psychiatric contexts. For example, terms such as “challenge” and “different” were particularly context dependent, as they could indicate either positive growth opportunities or negative stressors. In these cases, the authors reviewed the complete recorded dialogue and surrounding context to determine the emotional valence. Cultural nuances and adolescent-specific expressions were given special consideration, with disagreements resolved through detailed discussion until consensus was reached.

This systematic approach to sentiment classification, though qualitative in nature, ensured that ambiguous terms were consistently categorized, while respecting the complex emotional landscape of adolescents with psychiatric disorders participating in the virtual environment.

Any discrepancies were resolved through discussion and consensus. This rigorous process ensured a standardized, reproducible approach to sentiment categorization.

All available data were analyzed without imputation. No missing responses were reported for psychological measures, as participants completed and returned all self-reported questionnaires, as instructed. For qualitative responses, minor transcription gaps were resolved through researcher consensus, ensuring accurate thematic analysis. Due to the small sample size, inferential statistical analysis was not conducted.

### Ethics Approval

This study complied with the Declaration of Helsinki. Ethical approval was obtained from the Yokohama City University Ethics Committee (approval number F240600003). Detailed explanations of the study’s purpose, methods, potential risks, and benefits were provided to the participants and their legal guardians. Written informed consent was obtained from all participants, and the assent of minors aged 16 years or older with sufficient capacity for independent decision-making was also obtained.

The participants and their families provided explicit consent for appearing on the NHK television program *Project Aliens* through an agreement with the broadcaster. The research team only had access to transcriptions of the publicly broadcasted content from the program and did not have access to unedited interactions within the VR space. Additionally, the study used only predefined outcome measures, including primary and secondary outcomes. Personal data were anonymized using unique identification codes, ensuring confidentiality. The correspondence table linking codes to individual identities was securely stored at the research institution and was inaccessible to unauthorized personnel.

Participants were informed of their right to withdraw from the study at any time without consequences, and that their data would be excluded unless anonymized and aggregated. These measures ensured the protection of participant privacy, confidentiality of data, and compliance with ethical standards throughout the study.

In accordance with NHK’s standard practices for television production, participants received a modest honorarium as a token of appreciation for their cooperation in the program. This honorarium was not tied to their participation in the research study and was unrelated to the data collection or analysis. The amount was within the customary range for adolescent contributors in similar media contexts.

## Results

### Primary Outcome Measure

A case-by-case analysis of patient trajectories, based on structured evaluation, as shown in Table 1, revealed key psychological and social changes. Longitudinal changes in psychological status for the 3 adolescent participants. Data were collected at 3 time points: baseline (study registration), prebroadcast, and postbroadcast. Each participant’s subjective experiences and clinical observations were categorized into 4 domains: loneliness, resilience, depression (PRO), and psychiatrist observation. Psychiatrist observations were documented during routine clinical consultations at each time point.

**Table 1:** Longitudinal psychological changes in *Project Aliens* participants (N=3).

Participant and time points			Loneliness (PRO^a^)		Resilience (PRO)		Depression (PRO)		Psychiatrist observation
**Alien 1**									
	Baseline	Feeling disconnected when unable to talk to online friends		Distracted self with videos and games		No specific episode		Limited social engagement	
	Prebroadcase	Feeling excluded when unable to join conversations		Watched videos to change their mood		No specific episode		Limited social interaction skills	
	Postbroadcase	No specific episode		Found interacting with various people was meaningful and enriching		No specific episode		Developed better coping strategies and became more socially engaged	
**Alien 2**									
	Baseline	Feeling abandoned and lonely when a classmate stopped interacting after a seating change		No specific episode		Feeling overwhelmed by loneliness and sadness		Emotional distress, avoidance tendencies, and negative thought patterns	
	Prebroadcase	No specific episode		No specific episode		Feeling anxious about participating in the program		Anxiety about social interaction	
	Postbroadcase	“Talking with other participants who shared similar struggles helped me realize that I was not alone.”		“Expressing sadness and loneliness to others brought me relief.”		Feeling warmth from kind program staff, reducing isolation		Reduction in loneliness, improvement in coping skills, and alleviation of interpersonal distrust	
**Alien 3**									
	Baseline	“After missing school, pressure from my family to return caused tension, and I also became isolated from my friends.”		“Retreating to my room provided me with a sense of comfort.”		“However, I still felt distressed, as if I had no place to belong.”		Social isolation, depressive state, and avoidance behavior	
	Prebroadcase	“Continued school absence has resulted in prolonged feelings of isolation.”		“I tried going outside.”		“Feelings of depression and hopelessness persisted.”		Despite improvements in proactive coping strategies, the depressive symptoms remained.	
	Postbroadcase	“I started spending time and chatting with my friends after school.”		“I stopped avoiding tasks due to perfectionism and realized that taking action matters. Now, l have learned to accept my imperfections.”		“At first, I struggled to connect with my new classmates. Now I feel sad to part ways with them.”		Enhanced coping strategies, relief from depressive symptoms, and reduced feelings of isolation	

^a^PRO: patient reported outcome.

#### Patient-Reported Outcomes

The participants described changes in their experiences of loneliness, emotional responses, and coping mechanisms across the 3 time points (baseline, prebroadcast, postbroadcast).

Alien 1 initially reported feeling left out in social communication. Postbroadcast, he found the experience of talking to various people meaningful and enriching. From a psychiatrist’s observation, his social engagement had increased.

Alien 2 initially felt isolated and experienced sadness and loneliness. Postbroadcast, she was able to express her feelings directly to a friend, which provided relief. From a psychiatrist’s observation, postbroadcast, her loneliness decreased, coping skills improved, and interpersonal distrust was alleviated.

Alien 3 initially described feeling severe loneliness due to being unable to attend school and lacking communication with family. Postbroadcast, she reported positive social interactions, noting that she had been spending more time with friends. From a psychiatrist’s observation, her coping strategies improved, depressive symptoms were alleviated, and her feelings of isolation decreased.

### Secondary Outcome Measures

**Figure 2:**
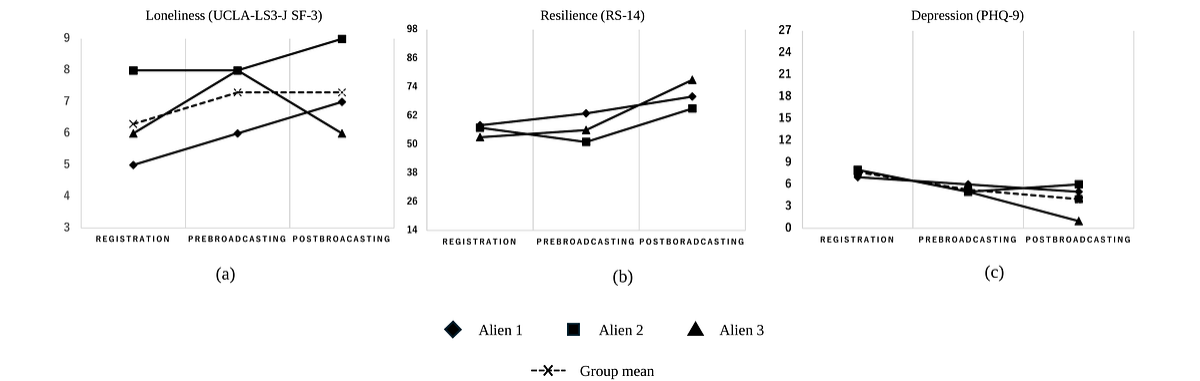
Longitudinal changes in psychological outcome measures. Visualization of individual trajectories and group means across 3 time points for (A) loneliness (UCLA-LS3-J SF-3, range 3-9), (B) resilience (RS-14, range 14-98), and (C) depression (PHQ-9, range 0-27). Each participant’s scores were measured at 3 time points: registration, prebroadcasting, and postbroadcasting. Alien 1 (diamond), alien 2 (square), and alien 3 (triangle) are represented by different markers. Group mean trends, indicated by × markers and dashed lines, illustrate the overall trajectory of changes in loneliness, resilience, and depressive symptoms among participants. PHQ-9: 9-item Patient Health Questionnaire; RS-14: 14-item Resilience Scale, short form; UCLA-LS3-J SF-3: 3-item Short-Form University of California, Los Angeles Loneliness Scale.

Figure 2 demonstrates changes in individual scores and group means across 3 psychological domains:

- Loneliness (UCLA-LS3-J SF-3): Scores increased slightly for alien 1 (+2) and alien 2 (+1), whereas alien 3 remained stable. This subtle increase in perceived loneliness may reflect heightened awareness of social needs following the program’s interpersonal engagement.
- Resilience (RS-14): Resilience improved across participants, with the largest increase observed in alien 3 (+24). This substantial improvement suggests enhanced psychological capacity to cope with adversity, particularly notable in alien 3, whose resilience score moved from below average to above average.
- Depression (PHQ-9): Depression scores decreased for all participants, with alien 3 showing the most significant reduction (−7). When interpreted according to established clinical severity categories (0-4: minimal; 5-9: mild; 10-14: moderate; 15-19: moderately severe; 20-27: severe), all participants showed clinically meaningful improvements. Alien 1 showed gradual improvement, while remaining in the mild category (7→6→5); alien 2 initially improved then slightly increased but stayed within the mild range (8→5→6); and alien 3 achieved the most significant change, transitioning from mild depression to minimal/none (8→5→1).

Each psychological measure exhibited different patterns of change across the 3 time points, as shown in Figure 2:

- Loneliness (UCLA-LS3-J SF-3): The group mean increased from 6.3 (SD 1.5) at registration to 7.3 (SD 1.2) prebroadcast and then remained stable at 7.3 (SD 1.5) postbroadcast.
- Resilience (RS-14): The group mean increased from 56.0 (SD 2.6) at registration to 56.7 (SD 6.0) prebroadcast, followed by a more substantial rise to 70.7 (SD 6.0) postbroadcast.
- Depression (PHQ-9): The group mean decreased from 7.7 (SD 0.6) at registration to 5.3 (SD 0.6) prebroadcast, and then further dropped to 4.0 (SD 2.6) postbroadcast.

#### Sentiment Analysis of Speech

The sentiment analysis, conducted as a secondary outcome measure, revealed distinct emotional dynamics across the program’s stages. Positive expressions dominated scene A, whereas scenes B and C reflected more balanced emotional expressions, indicating deeper emotional engagement.

**Figure 3:**
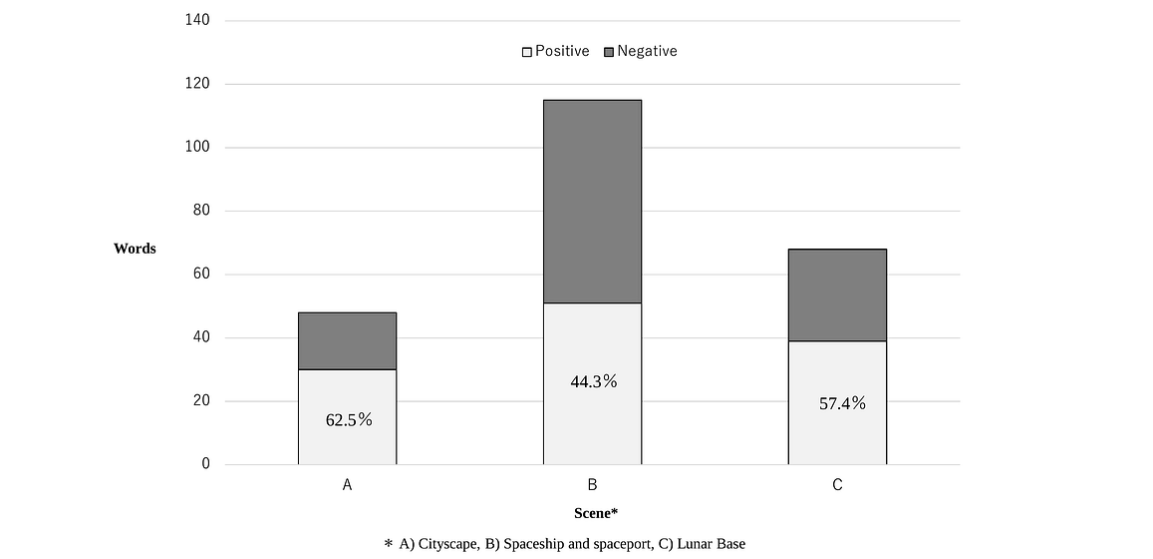
Quantitative analysis of emotional expression patterns. Temporal distribution of emotional expressions categorized by valence (positive/negative) across program stages. Frequencies represent word-level sentiment analysis of participant utterances. Scene (A) Cityscape shows predominantly positive expressions during initial interactions. Scene (B) Spaceship/Spaceport demonstrates increased emotional complexity with both positive and negative expressions. Scene (C) Moon reflects a shift toward resolution and hope. *Word frequency counts.

Figure 3 illustrates the sentiment distribution across different scenes, providing a visual representation of how the participants’ emotions varied during each scene of *Project Aliens*. Scene A contained a total of 48 emotional expressions: 18 (37.5%) negative and 30 (62.5%) positive. Scene B contained a total of 115 emotional expressions: 64 (55.7%) negative and 51 (44.3%) positive. Scene C contained a total of 68 emotional expressions: 29 (42.6%) negative and 39 (57.4%) positive.

In scene A, the participants frequently expressed positive emotions, such as excitement and curiosity, using terms such as “cute” and “fun.” These findings suggest that the design of the initial VR environment successfully engaged participants and reduced their apprehension. However, as the journey progressed to scene B, more complex emotional responses emerged. Participants used words such as “painful” and “anxious” while mentioning “courage” and “challenge,” indicating the difficulties they faced and their ability to confront them. By scene C, the focus shifted to closure, as reflected in expressions such as “finish” and “miss you,” and optimism, as reflected in expressions such as “hope” and “wish.”

#### Participant Dialogue Across Scenes

To illustrate the emotional progression observed in the sentiment analysis, representative participant dialogues from each scene are provided next.

In scene A (Cityscape), the participants introduced themselves through their avatars, using them as tools for self-expression and connection.

Hi, I’m Ramune. I chose a light-blue color that feels like “me.”

Alien 1

I’m Pichi. I wanted to get better at thinking before I speak, so I made my head look like a brain.

Alien 2

I’m Maruhachi. I put eyes in different places so I can see things from multiple perspectives.

Alien 3

In scene B (Spaceship and Spaceport), the participants began sharing deeper emotions, revealing vulnerabilities and finding validation in each other’s experiences.

Hearing others talk about their struggles makes me realize I’m not the only one who feels trapped sometimes.

Alien 3

When I have a panic attack, I can’t move or do anything. Having something comforting nearby really helps in those moments.

Alien 1

There’s a song lyric that really speaks to me: “All the things I can’t express just race past me with a face of sorrow.” I tend to bottle up my emotions, but I don’t have much capacity, so when it overflows, I just break down.

Alien 2

The final scene C (Lunar Base) reflected a shift toward self-acceptance and motivation for personal growth, with the participants expressing both lingering concerns and new resolutions.

I’ve been through a lot, but I feel like I’m finally looking forward. Maybe that’s the most important thing.

Alien 2

I think I need to trust myself a little more.

Alien 3

I still worry about going back to work or school. What if my panic attacks come back?

Alien 1

There’s no need to force change, but we should keep challenging ourselves. That’s how we take the first step.

Alien 3

## Discussion

### Principal Findings

This case series examined the psychological impact of *Project Aliens*, an innovative VR-based television program on Japanese public broadcasting, demonstrating its therapeutic potential for adolescents with mental health challenges. The program provided a unique social VR-based experience, offering a safe space for emotional expression and peer support through structured facilitation and immersive virtual environments. The findings indicate potential improvements in psychological well-being, including reduced loneliness and depressive symptoms and enhanced resilience. These improvements were observed in psychological measures, as well as PROs throughout the duration of the program, from recording through broadcast viewing. Social VR-based programs, such as *Project Aliens*, which create immersive spaces for dialogue, peer support, and emotional processing, may pave the way for new mental health interventions. Within this psychologically safe VR environment, the participants could openly express positive and negative emotions while sharing their recovery journey and adverse experiences through mutual empathy [12,13].

A key factor in the program’s efficacy was the structured facilitation of participant engagement. The program design—featuring warm colors, relatable visual elements, and playful alien avatars—fostered a sense of safety and accessibility, reducing communication barriers. Although realistic avatars can enhance trust in virtual environments [18], research suggests that nonhumanoid avatars, such as animal avatars, may promote self-disclosure and intimacy by reducing self-presentation anxiety [19]. This aligns with *Project Aliens*’ approach, where the alien avatars may have encouraged freer self-expression and deeper emotional dialogue. Online self-disclosure, although different from face-to-face interactions, has been recognized as a valuable preliminary step for highly anxious adolescents transitioning to offline communication [20].

This social VR-based program actively encouraged participant-led interactions, while ensuring their psychological safety. Previous studies have shown that online peer support groups enhance emotional regulation and coping strategies [21]. Additionally, structured VR-based learning environments have been found to boost engagement, self-efficacy, and emotional expression [22]. Likewise, virtual therapeutic spaces require structured facilitation to sustain meaningful interactions and maximize psychological benefits [23,24]. In *Project Aliens*, facilitation has been embedded into the program’s design, helping participants build trust and develop communication skills.

The quantitative findings indicate improvements in resilience and reductions in depressive symptoms among participants. Along with these measurable changes, the participants’ reflections provided valuable qualitative insights. Many reported feeling less isolated and more encouraged to face challenges positively. Although these subjective accounts were not part of formal outcome measures, they complemented the quantitative data, offering a broader understanding of the program’s impact. The structured VR environment facilitated collaborative engagement, mutual support, and sharing of emotional experiences, fostering a strong sense of solidarity among participants. Although this study did not directly measure changes in social attitudes, previous studies suggest that VR experiences can enhance interpersonal engagement and emotional connections [25,26].

Notably, improvements in resilience and other PROs following the program indicate that these transformations may extend beyond the virtual environment. This process aligns with the *Proteus effect* [27], a psychological phenomenon where individuals adopt behaviors and attitudes consistent with the characteristics and expectations of their digital avatars, often unconsciously altering their self-perception based on how they believe others perceive their virtual representation. As participants engaged with their avatars in *Project Aliens*, they likely internalized aspects of their virtual identities, reinforcing self-efficacy, emotional expression, and adaptive coping strategies. These factors may have contributed to the psychological improvements observed postbroadcast.

### Limitations

This study has several limitations. First, the small sample size and brief follow-up period limit the generalizability of findings and the ability to assess long-term outcomes. Additionally, the participants’ subjective reflections may introduce recall bias, affecting the accuracy of reported psychological changes. Furthermore, the unique nature of this intervention—a VR-based television program—may limit its applicability to broader adolescent psychiatric populations. Additionally, the participants’ responses may have been influenced by factors, such as feeling excited for participating in media, receiving attention from production staff, and feeling aware of being filmed for public broadcast. These media-related factors may have impacted psychological measures independently of the VR intervention.

All participants were VR novices, which helped minimize the influence of prior VR experience; however, individual differences in VR adaptability (eg, motion sickness, interface familiarity) may have affected engagement and the overall effectiveness of the intervention. Future studies should account for these variations by implementing standardized conditions, such as screening for VR tolerance and stratifying participants accordingly. Additionally, the participant selection process may have introduced bias. As the participants were selected by NHK producers based on thematic and logistical considerations rather than through random selection, this method could have influenced the study’s findings, limiting the generalizability of results.

### Future Direction

Despite these limitations, this study highlights the potential of VR-based group psychotherapy and day-care programs for young psychiatric patients. The structured facilitation and avatar-based interaction elements used in *Project Aliens* could address unresolved clinical challenges in various clinical settings.

The therapeutic framework of *Project Aliens* could potentially be adapted for conventional clinical practice through simplified implementations without the broadcasting component. Particularly valuable is the inherent structure of *Project Aliens* that allows viewers to observe social interactions between participants who maintain their anonymity. This format enables individuals with psychiatric disorders to interact through avatars while allowing observation by others. Similar to how *Project Aliens* viewers observed authentic interactions without compromising participant identities, this model could enable medical students, residents, mental health professionals, or even patient and family organizations to observe therapeutic exchanges, deepening understanding and reducing stigma toward psychiatric disorders.

Future research should use methodologically rigorous designs to isolate the specific therapeutic effects of VR elements. During our recording process, the involvement of television staff prior to filming undoubtedly provided positive stimulation for the young participants. By controlling conditions and establishing verification scenarios different from television program settings, while only changing the delivery medium, it would be possible to accurately evaluate the specific effects of the VR component. Additionally, if we could quantify neurological synchronization patterns related to empathy development and emotional regulation during avatar-mediated communication by collecting physiological data, such as electroencephalograms, heart rate variability, and oxytocin sampling, we might gain valuable insights into the mechanisms through which VR-based social interactions facilitate therapeutic change.

Through such validation processes, social VR could be refined as a structured therapeutic tool in psychiatric care, potentially creating new therapeutic support methods to expand mental health services for young people.

## Abbreviations

ASD: autism spectrum disorder
NHK: Nihon Housou Kyoukai
PHQ-9: 9-item Patient Health Questionnaire
PRO: patient-reported outcome
RS-14: 14-item Resilience Scale, short form
UCLA: University of California, Los Angeles
UCLA-LS3-J SF-3: 3-item Short-Form UCLA Loneliness Scale
VR: virtual reality

## Data Availability

The datasets generated and analyzed during the study are not publicly available due to privacy protection and the scope of the research plan but are available from the corresponding author upon reasonable request.

## References

[1] Fodor LA, Coteț CD, Cuijpers P, Szamoskozi S, David D, Cristea IA (2018). The effectiveness of virtual reality based interventions for symptoms of anxiety and depression: a meta-analysis. Sci Rep.

[2] Park MJ, Kim DJ, Lee U, Na EJ, Jeon HJ (2019). A literature overview of virtual reality (VR) in treatment of psychiatric disorders: recent advances and limitations. Front Psychiatry.

[3] Geraets CN, van der Stouwe EC, Pot-Kolder R, Veling W (2021). Advances in immersive virtual reality interventions for mental disorders: a new reality?. Curr Opin Psychol.

[4] Mosher MA, Carreon AC, Craig SL, Ruhter LC (2021). Immersive technology to teach social skills to students with autism spectrum disorder: a literature review. Rev J Autism Dev Disord.

[5] (2021). Survey on various issues in student guidance, such as problem behavior and school absenteeism among children and students. Ministry of Education, Culture, Sports, Science and Technology.

[6] (2022). NHK launches new VR documentary series Project Aliens. ITmedia.

[7] Radez J, Reardon T, Creswell C, Lawrence PJ, Evdoka-Burton G, Waite P (2021). Why do children and adolescents (not) seek and access professional help for their mental health problems? A systematic review of quantitative and qualitative studies. Eur Child Adolesc Psychiatry.

[8] Chmielowska M, Mannocci N, Tansel A, Zisman-Ilani Y (2022). Peer support and shared decision making in open dialogue: opportunities and recommendations. Front Psychol.

[9] Murphy R, Huggard L, Fitzgerald A, Hennessy E, Booth A (2024). A systematic scoping review of peer support interventions in integrated primary youth mental health care. J Community Psychol.

[10] Scanlon CL, Del Toro J, Wang M (2020). Socially anxious science achievers: the roles of peer social support and social engagement in the relation between adolescents’ social anxiety and science achievement. J Youth Adolesc.

[11] Lynch H, McDonagh C, Hennessy E (2021). Social anxiety and depression stigma among adolescents. J Affect Disord.

[12] Kenyon K, Kinakh V, Harrison J (2023). Social virtual reality helps to reduce feelings of loneliness and social anxiety during the COVID-19 pandemic. Sci Rep.

[13] Karami B, Koushki R, Arabgol F, Rahmani M, Vahabie A (2021). Effectiveness of virtual/augmented reality–based therapeutic interventions on individuals with autism spectrum disorder: a comprehensive meta-analysis. Front Psychiatry.

[14] Cho H Hikaru Cho's official website. Hikaru Cho.

[15] Arimoto A, Tadaka E (2019). Reliability and validity of Japanese versions of the UCLA loneliness scale version 3 for use among mothers with infants and toddlers: a cross-sectional study. BMC Womens Health.

[16] Nishi D, Uehara R, Kondo M, Matsuoka Y (2010). Reliability and validity of the Japanese version of the Resilience Scale and its short version. BMC Res Notes.

[17] Muramatsu K, Miyaoka H, Kamijima K, Muramatsu Y, Tanaka Y, Hosaka M, Miwa Y, Fuse K, Yoshimine F, Mashima I, Shimizu N, Ito H, Shimizu E (2018). Performance of the Japanese version of the Patient Health Questionnaire-9 (J-PHQ-9) for depression in primary care. Gen Hosp Psychiatry.

[18] Aseeri S, Interrante V (2021). The influence of avatar representation on interpersonal communication in virtual social environments. IEEE Trans Visual Comput Graphics.

[19] Ichikawa A, Ihara K, Kawaguchi I (2023). Investigation of how animal avatar affects users’ self-disclosure and subjective responses in one-on-one interactions in VR space.

[20] Towner E, Grint J, Levy T, Blakemore S, Tomova L (2022). Revealing the self in a digital world: a systematic review of adolescent online and offline self-disclosure. Curr Opin Psychol.

[21] McGill BC, Sansom-Daly UM, Wakefield CE, Ellis SJ, Robertson EG, Cohn RJ (2017). Therapeutic alliance and group cohesion in an online support program for adolescent and young adult cancer survivors: lessons from “Recapture Life". J Adolesc Young Adult Oncol.

[22] Chen C, Chang S, Hwang G, Zou D (2021). Facilitating EFL learners’ active behaviors in speaking: a progressive question prompt-based peer-tutoring approach with VR contexts. Interact Learn Environ.

[23] Redburn J, Hayes B (2024). Facilitators and barriers to “positive outcomes” from cognitive–behavioral therapy, according to young people: a thematic synthesis. J Clin Psychol.

[24] Aebersold M, Villarruel A, Tschannen D, Valladares A, Yaksich J, Yeagley E, Hawes A (2015). Using a virtual environment to deliver evidence-based interventions: the facilitator’s experience. JMIR Serious Games.

[25] Ichino J, Ide M, Yokoyama H, Asano H, Miyachi H, Okabe D (2022). "I've talked without intending to": self-disclosure and reciprocity via embodied avatars. Proc ACM Hum-Comput Interact.

[26] Nikolaou A, Schwabe A, Boomgaarden H (2022). Changing social attitudes with virtual reality: a systematic review and meta-analysis. Ann Int Commun Assoc.

[27] Yee N, Bailenson J (2007). The Proteus effect: the effect of transformed self-representation on behavior. Human Commun Res.

